# Knockout of syntaxin-4 in 3T3-L1 adipocytes reveals new insight into GLUT4 trafficking and adiponectin secretion

**DOI:** 10.1242/jcs.258375

**Published:** 2022-01-10

**Authors:** Hannah L. Black, Rachel Livingstone, Cynthia C. Mastick, Mohammed Al Tobi, Holly Taylor, Angéline Geiser, Laura Stirrat, Dimitrios Kioumourtzoglou, John R. Petrie, James G. Boyle, Nia J. Bryant, Gwyn W. Gould

**Affiliations:** 1 Department of Biology and York Biomedical Research Institute, University of York. Heslington, York YO10 5DD, UK; 2 Henry Wellcome Laboratory for Cell Biology, Institute for Molecular, Cellular and Systems Biology, College of Medical Veterinary and Life Sciences, University of Glasgow, Glasgow G12 8QQ, UK; 3 Department of Biology, University of Nevada Reno, 1664 N. Virginia Street, Reno, NV 89557, USA; 4 Strathclyde Institute for Pharmacy and Biomedical Sciences, 161 Cathedral Street, University of Strathclyde, Glasgow G4 0RE, UK; 5 Institute of Cardiovascular and Medical Sciences, University of Glasgow, Glasgow G12 8QQ. UK; 6 School of Medicine, Dentistry and Nursing, University of Glasgow, Glasgow G12 8QQ, UK

**Keywords:** GLUT4, SNARE, Syntaxin, Membrane trafficking

## Abstract

Adipocytes are key to metabolic regulation, exhibiting insulin-stimulated glucose transport that is underpinned by the insulin-stimulated delivery of glucose transporter type 4 (SLC2A4, also known and hereafter referred to as GLUT4)-containing vesicles to the plasma membrane where they dock and fuse, and increase cell surface GLUT4 levels. Adipocytokines, such as adiponectin, are secreted via a similar mechanism. We used genome editing to knock out syntaxin-4, a protein reported to mediate fusion between GLUT4-containing vesicles and the plasma membrane in 3T3-L1 adipocytes. Syntaxin-4 knockout reduced insulin-stimulated glucose transport and adiponectin secretion by ∼50% and reduced GLUT4 levels. Ectopic expression of haemagglutinin (HA)-tagged GLUT4 conjugated to GFP showed that syntaxin-4-knockout cells retain significant GLUT4 translocation capacity, demonstrating that syntaxin-4 is dispensable for insulin-stimulated GLUT4 translocation. Analysis of recycling kinetics revealed only a modest reduction in the exocytic rate of GLUT4 in knockout cells, and little effect on endocytosis. These analyses demonstrate that syntaxin-4 is not always rate limiting for GLUT4 delivery to the cell surface. In sum, we show that syntaxin-4 knockout results in reduced insulin-stimulated glucose transport, depletion of cellular GLUT4 levels and inhibition of adiponectin secretion but has only modest effects on the translocation capacity of the cells.

This article has an associated First Person interview with Hannah L. Black and Rachel Livingstone, joint first authors of the paper.

## Abstract (precis)

**Summary:** Syntaxin-4 knockout reduces insulin-stimulated glucose transport, depletes levels of cellular GLUT4 and inhibits secretion of adiponectin but only modestly affects the translocation capacity of the cells.

## INTRODUCTION

Adipocytes exhibit a range of membrane trafficking events linked to the regulation of energy metabolism and glucose homeostasis. Best characterised amongst these is the insulin-stimulated delivery of the glucose transporter solute carrier family 2 member 4 (SLC2A4, also known and hereafter referred to as GLUT4) to the surface (Gould et al., 2020; Klip et al., 2019) and the constitutive secretion of a range of hormones, known collectively as adipocytokines (Fasshauer and Blüher, 2015; Lehr et al., 2012). As perturbation in either of these processes can lead to metabolic dysfunction (Carvalho et al., 2005; Graham and Kahn, 2007; Kahn, 2019; Kumar et al., 2010), identifying the mechanisms involved in the regulation of their exocytosis (or trafficking) is an important research goal.

Membrane trafficking between subcellular compartments, including the plasma membrane involves well-conserved molecular families that operate across species. One notable example is the soluble N-ethylmaleimide-sensitive factor attachment protein receptor (SNARE) family of proteins (Wang et al., 2017). Members of this family associated with the target (t-SNARE) and vesicle (v-SNARE) membranes bind and assemble to form a complex that provides the energy to drive fusion (McNew et al., 2000; Weber et al., 2000). Cognate pairing of v- and t-SNAREs can drive fusion providing an impetus to identify those SNAREs involved in GLUT4 delivery to the cell surface (Bryant and Gould, 2011; Olson et al., 1997).

A large body of experimental evidence has indicated a key role for syntaxin-4 (Stx4, also known and hereafter referred to as Sx4) in GLUT4 trafficking. Introduction of anti-Sx4 into permeabilised adipocytes inhibited insulin-stimulated uptake of 2-deoxy-D-deoxyglucose (2DG) by ∼50%, whereas a non-specific antibody was without effect (Volchuk et al., 1996); similarly, glutathione S-transferase (GST) tagged to Sx4 (GST-Sx4) was found to inhibit the appearance of GLUT4 on plasma membrane lawns in 3T3-L1 adipocytes (Cheatham et al., 1996), an effect not observed with GST-Sx3 (Olson et al., 1997). A 50% inhibition of insulin-stimulated GLUT4 translocation was also observed after microinjection of peptides corresponding to amino acid residues 106-122 of Sx4 but not Sx1, suggesting that disruption of the interaction between Sx4 and other components of the cognate SNARE complex impairs GLUT4 translocation (Macaulay et al., 1997).

Homozygotic disruption of the Sx4 gene in mice is embryonically lethal, but heterozygous Sx4*^+/−^* mice exhibit an ∼50% diminution in whole-body glucose uptake; an effect which parallels the ∼50% reduction in skeletal muscle GLUT4 translocation (Yang et al., 2001). These data are strongly supportive of a role for Sx4 in GLUT4 translocation within skeletal muscle, but it is of interest that Sx4*^+/−^* mice exhibited normal insulin-stimulated glucose transport in adipocytes despite a 50% reduction in Sx4 protein levels (Yang et al., 2001). This is unlikely to be explained by an excess of Sx4, as studies in 3T3-L1 adipocytes and cardiomyocytes indicate that Sx4 is expressed at levels broadly similar to those of other SNARE components identified as being involved in GLUT4 vesicle fusion with the cell surface (VAMP2 and SNAP23) (Bowman et al., 2019; Hickson et al., 2000). Further support for an important role for Sx4 is provided by studies of transgenic mice overexpressing Sx4 (Spurlin et al., 2004). These mice exhibit enhanced skeletal muscle glucose transport but did not display significantly increased glucose uptake in adipose tissue. Using siRNA to knock down VAMP2, SNAP23 and Sx4 in 3T3-L1 adipocytes reduced insulin-stimulated glucose transport by ∼50% in 3T3-L1 adipocytes (Kawaguchi et al., 2010). This study did suggest a requirement for Sx4 in tethering GLUT4-containing vesicles at the cell surface, but this was not assessed in living cells (Kawaguchi et al., 2010). A role for Sx4 is also supported by numerous studies indicating that Sx4 and its cognate, the Sec1/Munc18 (SM) family protein Munc18c (officially known as Stxbp3), play pivotal roles in the regulation of GLUT4 delivery to the cell surface (D'Andrea-Merrins et al., 2007; Kanda et al., 2005; Kioumourtzoglou et al., 2014; Oh et al., 2005; Tamori et al., 1998; Thurmond and Pessin, 2000; Thurmond et al., 1998, 2000). Collectively, these data support a role for Sx4 in GLUT4 translocation, particularly in skeletal muscle.

Many of the studies mentioned above exhibited only partial inhibition (Cheatham et al., 1996; Kawaguchi et al., 2010; Olson et al., 1997; Volchuk et al., 1996); this may be attributed to limitations in experimental systems and/or assay sensitivity, but the possibility that other mechanism(s) exist and act in parallel or in a compensatory fashion has not been addressed. The notable distinctions in the behaviour of adipose and muscle tissue in transgenic mice underscore the need to consider this possibility (Spurlin et al., 2004; Yang et al., 2001).

There is evidence that some SNARE-mediated trafficking events utilise functionally redundant SNAREs (Liu and Barlowe, 2002). Intriguingly, studies investigating the role of the v-SNARE VAMP2 in insulin-stimulated GLUT4 translocation revealed unexpected plasticity (Zhao et al., 2009). Simultaneous disruption of VAMP2, VAMP3 and VAMP8 completely block insulin-stimulated GLUT4 translocation in 3T3-L1 adipocytes; however, surprisingly this block could be overcome by individual re-expression of either VAMP2, VAMP3 or VAMP8 (Zhao et al., 2009). Such data prompted a re-evaluation of the role of Sx4 in GLUT4 translocation, particularly with the advent of genome editing and the ability to sensitively assay GLUT4 trafficking by using epitope-tagged reporters. Because complete Sx4 knockout in mice is lethal (Yang et al., 2001), we undertook genome editing to knock out Sx4 in 3T3-L1 adipocytes to test the hypothesis that Sx4 is not the only plasma membrane t-SNARE involved in GLUT4 translocation. We found that Sx4-knockout cells do not exhibit a change in insulin sensitivity but exhibit a reduced rate of insulin-stimulated 2DG transport, which can be accounted for by reduced total levels of cellular GLUT4. A similar reduction in adiponectin secretion was also observed. Kinetics analysis of translocation of ectopically expressed haemagglutinin (HA)-tagged GLUT4 conjugated to GFP (HA-GLUT4-GFP) indicates that, whereas loss of Sx4 decreases the maximal rate of GLUT4 trafficking, Sx4 depletion is not rate-limiting for GLUT4 delivery to the plasma membrane. Therefore, we conclude that a further mechanism, in addition to the one via Sx4, can mediate GLUT4-vesicle fusion with the plasma membrane of adipocytes but that Sx4 plays a key role in the maintenance of cellular GLUT4 levels and in adiponectin secretion.

## RESULTS

### Generation of Sx4-knockout cells

**Fig. 1.**
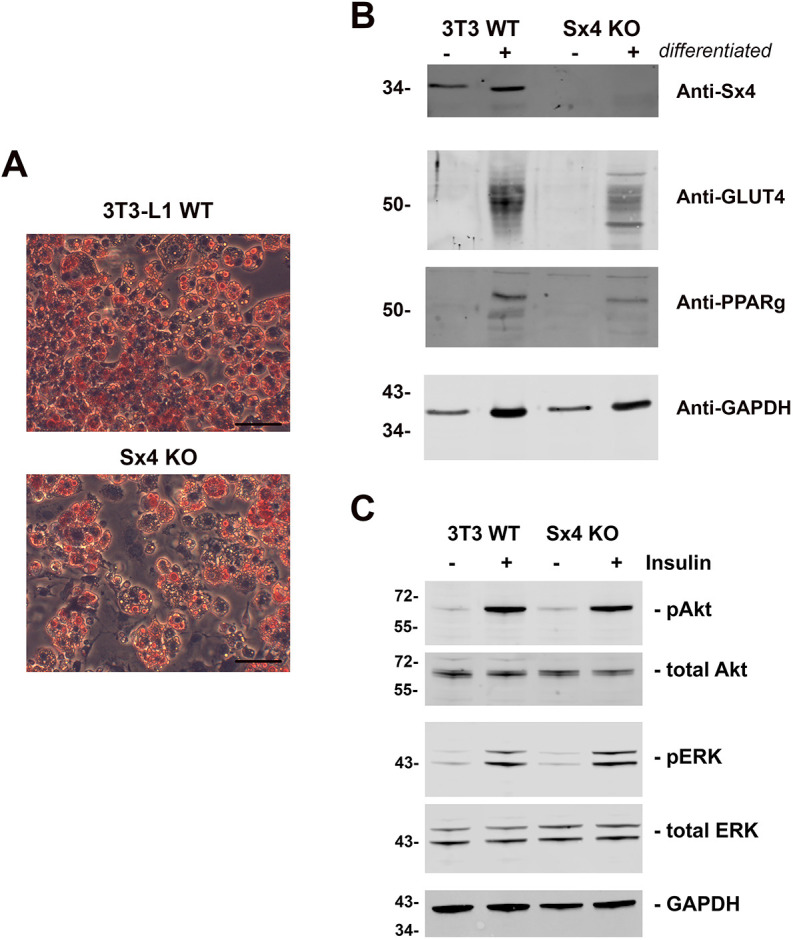
**Characteristics of Sx4-knockout 3T3-L1 cells.** Wild-type (WT) and Sx4-knockout (KO) 3T3-L1 fibroblasts were treated with adipocyte differentiation medium for 12 days. (A) Oil Red O staining of cells 12 days after differentiation. Scale bars: 150 μm. (B) Representative immunoblots, showing the expression of adipogenesis markers peroxisome proliferating factor (PPARγ) and GLUT4, which were increased following differentiation (+) in both parental (3T3 WT) and Sx4-KO lines. GAPDH is shown as a control, Sx4 confirms the validity of the knockout lines. 15 µg of protein was loaded on each lane. (C) Comparison of basal (-) and insulin-stimulated (+) signalling proteins in parental (3T3 WT) and Sx4-KO adipocytes at day 10. Cells were incubated in serum-free medium for 2 h prior to incubation with 100 nM insulin for 15 min. Lysates were immunoblotted for the indicated proteins. Data from a typical experiment are shown, repeated three times with qualitatively similar results; each lane contains 15 µg of protein. pAKT, phosphorylated Akt proteins; pERK, phosphorylated ERK proteins.

**Fig. 2.**
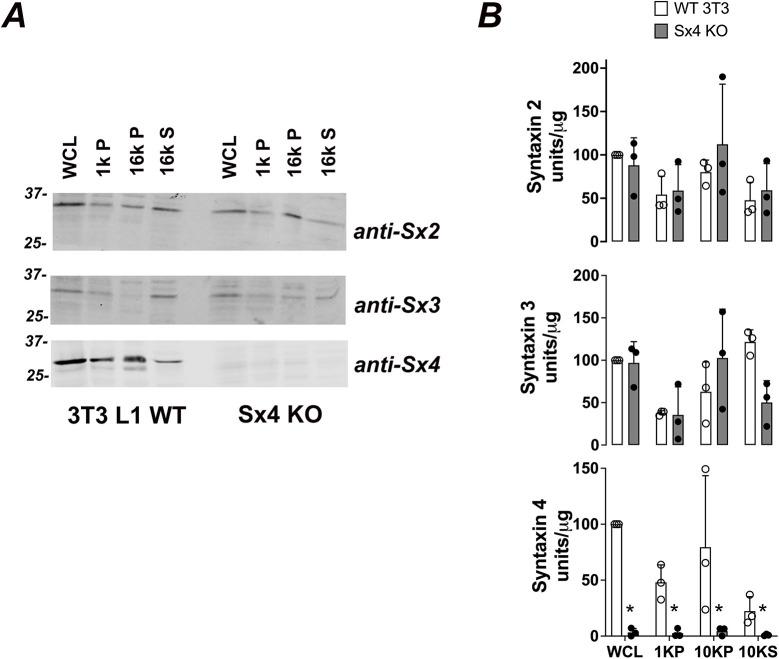
**t-SNARE expression in Sx4-knockout adipocytes.** (A) Representative immunoblots of subcellular fractions from either wild-type (WT) or Sx4-knockout (KO) 3T3-L1 adipocytes probed for the plasma membrane syntaxins (anti-Sx2, anti-Sx3 and anti-Sx4). WCL, whole-cell lysate. 1k P indicates the pellet obtained after centrifugation at 1000 ***g***, enriched for large debris and containing some of the plasma membrane; 16k P indicates the pellet obtained after centrifugation at 16,000 ***g***, containing heavy microsomes and plasma membrane; 16k S indicates the corresponding supernatant of 16k P, containing light microsomes and enriched in GLUT4 storage compartments (GSCs). Equal amounts of protein per fraction were loaded on each lane. (B) Quantification of separate immunoblots of the type shown in A from three biological replicates comparing wild-type 3T3 L1 and Sx4-knockout cells. No differences were observed in levels of Sx2 or Sx3 in any fraction. Sx4 protein levels were significantly reduced in all fractions. Data are presented as mean+s.d. *P<0.02 for all fractions (two-way ANOVA).

We used genome editing to knock out Sx4 in 3T3-L1 fibroblasts derived from mouse embryo fibroblasts. Multiple lines of Sx4-knockout cell lines were established and knock out of Sx4 was confirmed by immunoblot analysis. DNA sequence analysis of two independent clones confirmed frameshift mutations, one after amino acid 23, the other at residue 24 of Sx4. Regarding all experiments described in the following, at least three biological replicates were performed on one clone and those data were confirmed by using a second, independent clone. We found that Sx4-knockout cells differentiated into adipocytes, as evidenced by the accumulation of visible lipid droplets, expression of adipocyte-specific marker proteins – including PPARγ (Fig. 1A), and by Oil Red O staining (Fig. 1B). Furthermore, we observe robust insulin-stimulated phosphorylation of Akt and ERK proteins in Sx4-knockout cells at levels comparable to those observed in wild-type cells (Fig. 1C). Analysis of plasma membrane-associated SNARE proteins confirmed the absence of Sx4 in these cells, without changes in the levels or distribution of either Sx2 or Sx3 among the different membrane fractions analysed (Fig. 2).

### Sx4-knockout cells exhibit reduced insulin-stimulated glucose transport and decreased GLUT4 levels

**Fig. 3.**
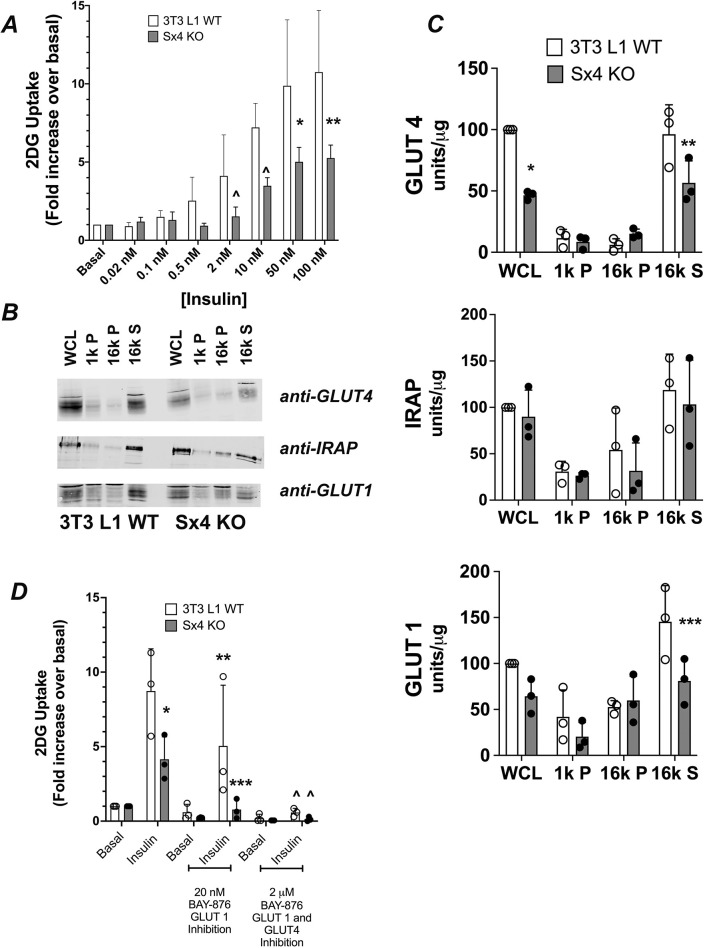
**Insulin-stimulated glucose transport in Sx4-knockout cells.** (A) Triplicate wells of cells treated with or without the indicated concentration of insulin for twenty minutes were used to assess uptake of 2-deoxy-D-glucose (2DG) (see Materials and Methods). Data were corrected for non-specific cellular isotope uptake by performing parallel assays in the presence of 10 µM cytochalasin B; data were then normalised to those obtained in the absence of insulin (basal) for each data set. Shown are the fold increases in uptake of 2DG at the indicated insulin concentrations, expressed as the mean+s.d. of at least three biological replicates. A significant difference between wild-type (WT) and Sx4-knockout (KO) cells was observed (*P=0.01 at 50 nM and **P=0.004 at 100 nM), and a trend towards significance seen at 2 nm and 10 nM (^*P*∼0.09). Basal rates of 2DG uptake were not different between wild-type and Sx4-knockout cells. Results were analysed by two-way ANOVA. (B) Representative immunoblots of subcellular fractions (as defined for Fig. 1) probed for GLUT4, GLUT1 and IRAP; equal amounts of each fraction were loaded per lane. (C) Quantification of at least three independent subcellular fractionation experiments quantified as described. Data are presented as mean+s.d. Results were analysed by two-way ANOVA. *P=0.001, **P=0.02, ***P=0.02. The difference in GLUT1 levels compared with whole-cell lysate (WCL) was not statistically significant (*P*=0.16). 1k P indicates the pellet obtained after centrifugation at 1000 ***g***, enriched for large debris and containing some of the plasma membrane; 16k P, indicates the pellet obtained after centrifugation at 16,000 ***g***, containing heavy microsomes and plasma membrane; 16k S indicates the corresponding supernatant of 16k P, containing light microsomes and enriched in GLUT4 storage compartments (GSCs). (D) Effect of the GLUT-specific inhibitor BAY-876 on 2DG uptake in non-stimulated (basal) and insulin-stimulated (100 nM) cells. Insulin-stimulated 2DG uptake was significantly reduced in Sx4-knockout cells compared with that in wild-type cells (*P=0.006). In the presence of 20 nM BAY-876, insulin-stimulated 2DG uptake in wild-type cells was inhibited by 42% (**P=0.05). However, 20 nM BAY-876 almost completely abolished insulin-stimulated 2DG uptake in Sx4-knockout cells (***P=0.013) compared to insulin-stimulated 2DG uptake in Sx4-knockout cells in the absence of BAY-876. Notice that – in this cell type – 2DG uptake was not significantly different to basal uptake (i.e. in cells not stimulated with insulin). Upon treatment with 2 µM BAY-876, 2DG uptake was essentially completely abolished in either cell line (^*P*∼0.03) compared to corresponding levels of 2DG uptake in the absence of BAY-876). Data are presented as mean+s.d. Statistical analysis was performed using two-way ANOVA in Prism; data from three independent experiments were analysed using WT and KO cells.

We assayed insulin-stimulated glucose transport in wild-type control and Sx4-knockout cells (Fig. 3A). As shown, Sx4-knockout cells exhibited a reduction in 2DG uptake. Wild-type cells stimulated with 100 nM insulin exhibited a 10.7±3.9-fold increase in 2DG uptake, whereas Sx4-knockout cells stimulated with insulin at the same concentration exhibited only a 5.3±0.8-fold increase. Similar reductions were observed at lower insulin concentrations (see Fig. 3A), but stimulation with 50 nM or 100 nM insulin was not significantly different between control and Sx4 wild-type cells, indicating that the effect was not a consequence of impaired insulin signalling. No difference in basal (unstimulated) rates of 2DG uptake was observed between wild-type and Sx4-knockout cells. The half-time of the rate of increase in insulin-stimulated 2DG uptake at 100 nM insulin was unchanged upon knockdown of Sx4 (not shown but see below for further examination of this point). Sx4 knockdown was associated with a 54±3% reduction of total GLUT4 content (Fig. 3B,C). This reduction was found mainly in the 16,000 ***g*** supernatant (16k S), a fraction known to be enriched in the GLUT4 storage compartments (GSCs) and insulin-responsive GLUT4-containing vesicles (IRVs) that translocate to the cell surface in response to insulin. Similar decreases in GLUT1 levels in this fraction were noticed. By contrast, levels of insulin-responsive aminopeptidase (IRAP) were unchanged. This suggests that the reduction in insulin-stimulated 2DG can be explained by a reduction in GLUT4 levels. This was confirmed using the GLUT-specific inhibitor BAY-876. At low concentrations (∼20 nM), this compound is selective for GLUT1; at higher concentrations (2 µM), both GLUT1 and GLUT4 are inhibited. Fig. 3D confirms that, in wild-type 3T3-L1 adipocytes, 43±24% of insulin-stimulated 2DG uptake is inhibited by 20 nM BAY-876 suggesting that 60% of insulin-stimulated glucose transport in wild-type cells is GLUT4-dependent. In Sx4-knockout cells, insulin-stimulated 2DG uptake is essentially inhibited 85±16% in the presence of 20 nM BAY-876, a result consistent with the hypothesis that, in Sx4-knockout cells, the majority of insulin-stimulated 2DG uptake is GLUT1 dependent.

**Fig. 4.**
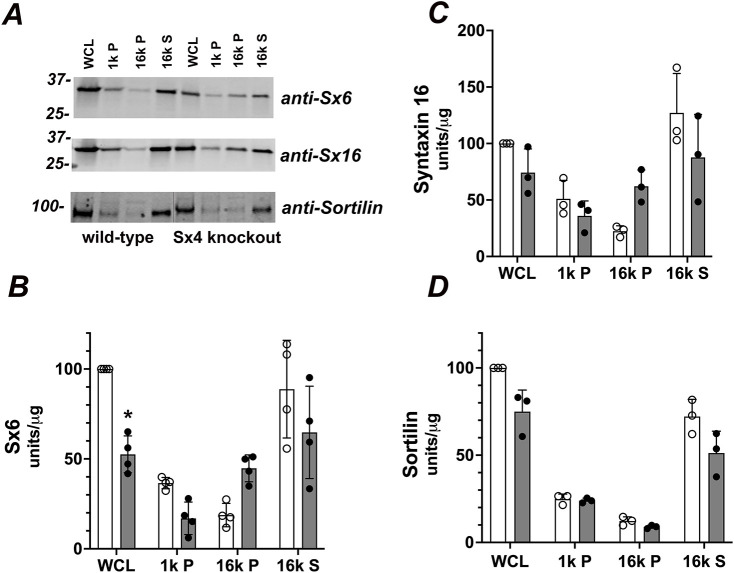
**Protein levels of Sx6, 16 and sortilin are unchanged in Sx4-knockout cells.** (A) Representative immunoblots of subcellular fractions (as described in Fig. 1) from a single set of wild-type or Sx4-knockout 3T3-L1 adipocytes probed for the indicated proteins. Equal amounts of each fraction were loaded. Notice that in the anti-sortilin blot, an empty lane has been removed (indicated by the black vertical line) to facilitate comparison with other immunoblots from this data set. (B–D) Quantification of separate immunoblots of this type from three separate platings of cells are shown in B (Sx6) C (Sx16) and D (sortilin). Differences between wild-type and Sx4-knockout cells did not reach significance for any fraction/protein, except for a reduction of Sx6 in Sx4-knockout cells compared to that in wild-type cells (*P=0.001 in B). Data are presented as mean+s.d. Results were analysed by two-way ANOVA.

Previous work from our group has shown that knockdown of Sx6 or Sx16 abrogates GLUT4 sorting into IRVs and results in a decrease of total GLUT4 levels within the cell (Perera et al., 2003; Proctor et al., 2006). Similarly, an important role in GLUT4 trafficking has also been proposed for sortilin (Huang et al., 2013; Pan et al., 2017, 2019). We, therefore, examined the levels of these proteins to test the hypothesis that knockout of Sx4 had in some way altered the machinery required for GLUT4 sequestration into GSCs/IRVs. Fig. 4 shows that neither the levels nor the distribution of Sx16 or sortilin are altered in Sx4-knockout cells compared to wild-type cells. A small reduction in total levels of Sx6 was observed in whole-cell lysates, but analysis of the distribution of this SNARE among the subcellular fractions did not reveal any significant changes.

### Adiponectin secretion is impaired in Sx4-knockout cells

**Fig. 5.**
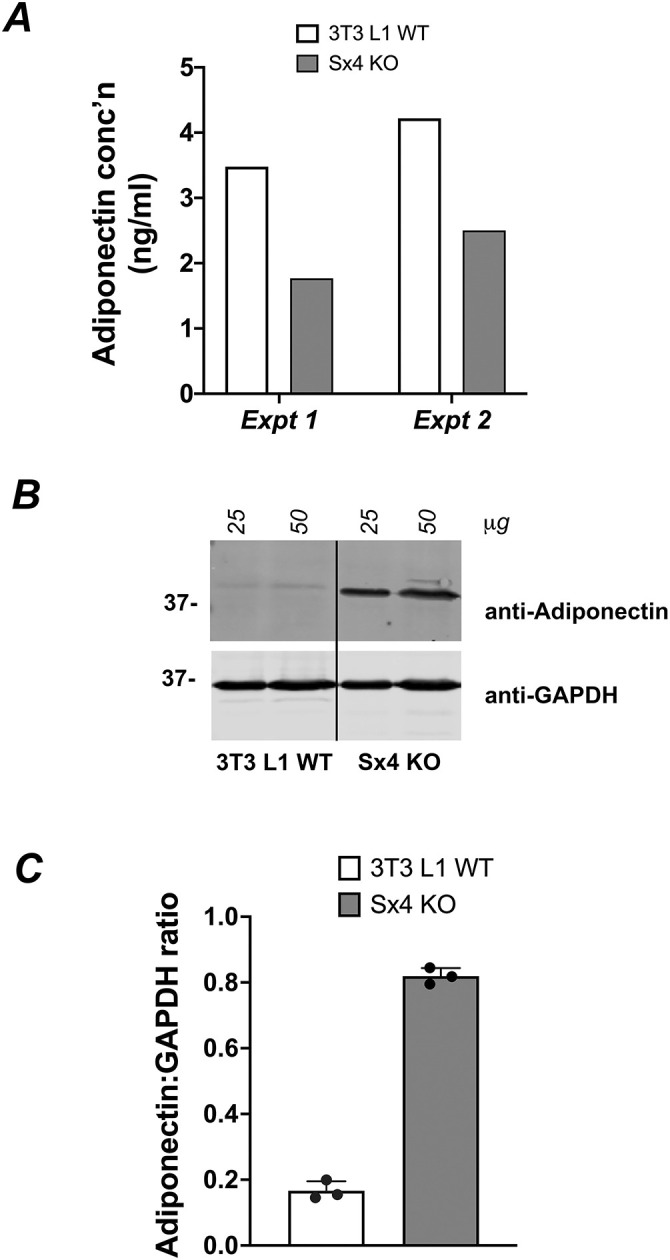
**Adiponectin secretion is impaired in Sx4-knockout cells.** (A) Results of ELISA assay measuring adiponectin released from wild-type or Sx4-knockout cells during 24 h incubation in serum-free medium. Data from two independent assays are shown, each value representing triplicate measurements under each condition. Notice that multiple assays were performed using different volumes of supernatant to ensure linearity and reproducibility; however, a single point is shown here for clarity. (B) Either wild-type or Sx4-knockout cells were incubated in serum-free medium for 24 h and cell lysates were prepared. Lysates were immunoblotted for adiponectin or GAPDH as indicated; either 25 or 50 µg of protein was loaded per lane. Notice that the blot shown had irrelevant lanes removed (indicated by the vertical black line). (C) Quantification of three experiments. Data are presented as mean+s.d. The difference in adiponectin-to-GAPDH ratio in Sx4-knockout and wild-type cells was significant (*P*=0.001; two-way ANOVA).

3T3-L1 adipocytes secrete a range of adipocytokines via the delivery of secretory cargo to the cell surface. We, therefore, tested whether Sx4 knockout affected adiponectin secretion. To quantify this, we used an adiponectin ELISA assay and found, in two separate platings of cells, that secretion of this protein was reduced by 50% upon Sx4 knockout (Fig. 5A). This was accompanied by increased total cell adiponectin levels, as revealed by immunoblot analysis (Fig. 5B,C). Thus, the reduction in adiponectin secretion seems to arise from defective exocytosis of the protein rather than an effect on its expression or stability.

### HA-GLUT4-GFP recycling reveals that the translocation machinery is largely intact

**Fig. 6.**
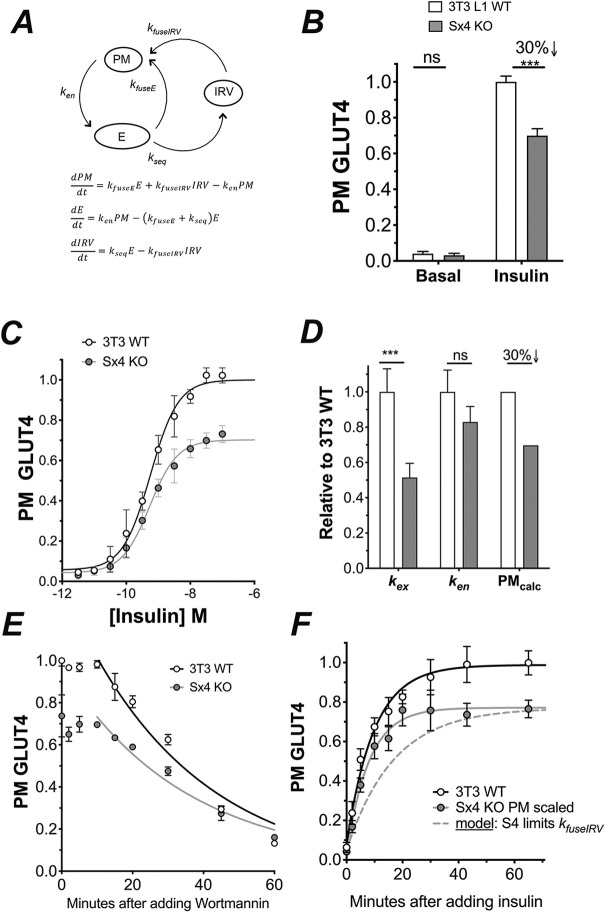
**Recycling of ectopic HA-GLUT4-GFP in Sx4-knockout cells.** (A) Schematic of the model of GLUT4 recycling used, with GLUT4 trafficking between the plasma membrane (PM), endosomes (E), and GSCs and/or IRVs (IRV) (Habtemichael et al., 2011; Muretta et al., 2008). The rate constants for each step are indicated. (B) GLUT4 levels at the PM in the presence and absence of insulin (100 nM, 45 min). There is a 30% reduction in cell surface GLUT4 levels in Sx4-knockout cells compared to those in control cells; the mean+s.d. of six independent experiments is shown (***P<0.001 by two-way ANOVA). No significant (n.s.) effect on basal levels was observed. (C) Effect of insulin on cell-surface GLUT4 levels. EC_50_ values of control vs Sx4-knockout cells are 0.54 vs 0.57 nM, respectively (data from four independent experiments; mean±s.d.). (D) Kinetics parameters determined as outlined in Materials and Methods assayed in control and Sx4-knockout cells. Data are presented as mean+s.d. Observed was a statistically significant 50% decrease in *k_ex_* (***P=0.004 by non-linear fit analysis) but no effect on *k_en_*. When fitted to the model shown in A, prediction is a 30% decrease in GLUT4 levels upon insulin stimulation at the PM in Sx4-knockout cells relative to control cells (PM_calc_). (E) Time course of the decline in PM GLUT4 levels upon wortmannin (100 nM) addition; cells were pre-treated with insulin for 45 min wortmannin added for the times shown before assay of cell surface GLUT4. The rate constant of endocytosis, *k_en_*, was unchanged (mean±s.d. of *n*=4 independent experiments). (F) Half-time of the increase in relative surface GLUT4 in response to insulin; this was not significantly changed in Sx4-knockout cells relative to wild-type cells (mean±s.d. of *n*=4 independent experiments). The dashed line shows the result predicted if Sx4 is rate limiting for fusion of IRVs with the PM. Similar levels of HA-GLUT4-GFP were expressed in both wild-type and Sx4-knockout cells as judged by GFP fluorescence levels (not shown).

Our data indicate that Sx4-knockout cells exhibit reduced rates of insulin-stimulated glucose transport via a mechanism involving a reduction in total cell GLUT4 levels (discussed further below). We, therefore, decided to examine the effect of GLUT4 re-expression in these cells by using the well-characterised HA-GLUT4-GFP (Muretta et al., 2007, 2008) and analysing the kinetics of its trafficking by using the model shown in Fig. 6A (Habtemichael et al., 2011; Muretta and Mastick, 2009; Muretta et al., 2008). In the experiments that follow, expression levels of HA-GLUT4-GFP were routinely too low for easy quantification using immunoblotting; however, these conditions were chosen to minimise potential overexpression artefacts (Brewer et al., 2019; Habtemichael et al., 2011; Muretta et al., 2007, 2008). The data obtained are shown in Fig. 6, where experiments in Sx4-knockout cells were compared to those in wild-type 3T3-L1 adipocytes expressing similar amounts of HA-GLUT4-GFP – as judged by GFP intensity. Fig. 6B shows that Sx4-knockout cells exhibit a 30% reduction in maximal levels of insulin-stimulated HA-GLUT4-GFP at the plasma membrane. In both WT and Sx4 KO cells was a robust translocation of GLUT4 in response to insulin (25-fold and 22-fold, respectively) relative to basal GLUT4 levels in the two cell lines. This was not accompanied by an alteration in the EC_50_ of the insulin response (Fig. 6C; 0.54 and 0.57 nM, respectively), consistent with the transport data shown in Fig. 3. Assay of the kinetics of GLUT4 recycling revealed that this can largely be explained by a 50% reduction in the rate constant of exocytosis *k_ex_* (Fig. 6D). To test this, we assayed wortmannin-driven endocytosis of surface-labelled HA-GLUT4-GFP, the rate of which was found to be identical between wild-type and Sx4-knockout cells (Fig. 6E). These measured rate constants – when fitted to the model shown in Fig. 6A – predict a 30% decrease in insulin-stimulated GLUT4 levels at the plasma membrane of Sx4-knockout cells relative to those observed in wild-type cells (Fig. 6F). Interestingly, examination of the time course of basal transition (i.e. in cells not stimulated with insulin) versus insulin-stimulated transition provides further insights (Fig. 6F): the half-time of the increase in relative surface GLUT4 was not significantly changed in Sx4-knockout cells relative to that in wild-type cells (Fig. 6F), consistent with the transition time course for insulin-stimulated glucose transport (data not shown). This finding is in marked contrast to that predicted if Sx4 is, indeed, rate limiting for the fusion of IRVs to the plasma membrane in response to insulin (dashed line in Fig. 6F) and suggests that the observed decrease in GLUT4 exocytosis in Sx4-knockout cells occurs at a different intracellular trafficking step. This is discussed further below.

## DISCUSSION

### Sx4 knockout perturbs GLUT4 levels and impairs secretion

There are considerable experimental data supporting a role for Sx4 in insulin-stimulated delivery of GLUT4 to the plasma membrane of muscle cells and adipocytes (Kawaguchi et al., 2010; Kioumourtzoglou et al., 2014; Olson et al., 1997; Tamori et al., 1998; Volchuk et al., 1996; Widberg et al., 2003; Yang et al., 2001). It is noteworthy that many of these studies revealed that a significant fraction of insulin-stimulated glucose transport and/or GLUT4 translocation remained after inhibition/knockdown of Sx4, hinting other mechanisms might also be involved in this process. To address this, we generated Sx4-knockout 3T3-L1 cell lines and found that they exhibit a 50% reduction in insulin-stimulated glucose transport compared to that in wild-type cells but with an unchanged EC_50_ for insulin (Fig. 3A). We found that this effect can largely be explained by a corresponding reduction in GLUT4 levels. It is perhaps worth noting here that a previous study of Sx4 knockdown in adipocytes did not examine this point (Kawaguchi et al., 2010). Using a simple fractionation procedure, we observed that this reduction was largely confined to a fraction known to be enriched in GSCs/IRVs (Fig. 3B,C). Using the GLUT-selective inhibitor BAY-876, we showed that in Sx4-knockout cells, the overwhelming majority of insulin-stimulated 2DG uptake is inhibited by 20 nM BAY-876, compared to just 40% in wild-type cells (Fig. 3D). At this concentration, BAY-876 is GLUT1 selective (Siebeneicher et al., 2016), hence these data argue that, upon Sx4 knockout, GLUT1 is the major transporter active in these cells, consistent with the reduction of GLUT4 levels in the GSC/IRV-enriched fraction. Strikingly, the levels and distribution of IRAP and Sx16/Sx6 are unchanged upon Sx4 knockdown (Fig. 4). These proteins play important roles in the trafficking of GLUT4 into IRVs (Gould et al., 2020; Perera et al., 2003; Proctor et al., 2006; Ross et al., 1998; Shewan et al., 2003; Watson et al., 2008; Yeh et al., 2007), suggesting that at least some components of the machinery for this remain largely intact. This will be discussed further below.

Adipocytes secrete many small adipocytokines that act in an autocrine and paracrine fashion to regulate whole-body energy metabolism (Kahn, 2019; Minokoshi et al., 2003). We observed a significant reduction in adiponectin secretion in Sx4-knockout cells, with an accompanying increase in total cell adiponectin levels (Fig. 5). These observations are consistent with a reduction of exocytic events for secretory cargo in cells lacking Sx4. These data are, to our knowledge, the first to implicate Sx4 in adiponectin secretion; effects on other adipocytokines, such as leptin or adipsin, will be worthwhile to assess in future experiments to ascertain whether this is a global (all adipocytokines) or protein-selective effect. It is again noteworthy that the reduction in secretion is only partial, suggesting that compensatory/alternative pathways for delivery of adiponectin-containing vesicles to the plasma membrane operate in these cells. Nevertheless, these data argue that Sx4 plays multiple roles in adipocyte biology.

### Ectopic GLUT4 translocation in the absence of Sx4

To dissect further the role of Sx4 in GLUT4 trafficking, we used ectopic expression of HA-GLUT4-GFP; the presence of GLUT at the cell surface can be quantified by antibodies against the HA-epitope (present in the exofacial loop of GLUT4) and total GLUT4 levels assessed via the GFP tag (Blot and McGraw, 2006; Habtemichael et al., 2011; Karylowski et al., 2004; Martin et al., 2006; Muretta et al., 2007, 2008). Here, we found that maximal insulin-stimulated GLUT4 translocation was only modestly impaired in Sx4-knockout cells (Fig. 6B) and that the EC_50_ was unchanged (Fig. 6C) compared to that in wild-type cells expressing comparable levels of HA-GLUT4-GFP. Thus, despite the complete absence of Sx4, a substantial translocation of GLUT4 was observed (typically >22-fold compared to 25-fold in control cells; Fig. 6B), indicating that these cells have machinery capable of delivering GLUT4 to the plasma membrane in response to insulin. This conclusion is further supported by our observation that IRAP exhibits robust insulin-stimulated translocation in Sx4-knockout cells, as assessed by subcellular fractionation (Fig. S1). We used the HA-epitope to further examine the recycling properties of GLUT4 in these cells and interpret the results within the framework of an established recycling model shown in Fig. 6A (Habtemichael et al., 2011; Muretta et al., 2007, 2008). These analyses revealed that Sx4 knockout is accompanied by a 50% reduction in the rate constant for exocytosis *k_ex_*, with no effect on the endocytic *k_en_* (Fig. 6D). Examination of basal versus insulin-stimulated transition kinetics (Fig. 6F) revealed that, although there was significant inhibition of *k_ex_*, the phenotype of these cells did not match the one predicted when Sx4 was rate-limiting for the final fusion step between GLUT4-containing and the plasma membrane (see Fig. 6F, *k_fuseIRV_*). Hence, our data revealed that GLUT4 is able to readily access the plasma membrane in response to insulin, albeit with a slower rate of exocytosis.

There are several potential explanations for this. First, many studies have indicated a degree of plasticity in SNARE protein function where loss of one SNARE can be compensated for by the system using a different SNARE, or where multiple SNAREs can perform similar functions. This has been described for the v-SNAREs in GLUT4 translocation (Sadler et al., 2015; Zhao et al., 2009) and in the exocytosis of chromaffin granules in neuroendocrine cells (Borisovska et al., 2005). In some cases, loss of one SNARE is accompanied by a compensatory upregulation of a related SNARE, e.g. loss of Sec22p in yeast is compensated for by upregulation of Ykt6p (Liu and Barlowe, 2002). This does not appear to be the case here, as levels of Sx2, Sx3, Sx16 and Sx6 are unchanged. However, whether the machinery that normally regulates Sx4 assembly into SNARE complexes can ‘hijack’ Sx2 or Sx3 remains to be explored. Recent work argues that the assembly of a functional SNARE complex by cognate SNARE interactions is a point of convergence for many regulatory mechanisms, including SM family proteins, tethering complexes, Rab proteins, etc. (Baker and Hughson, 2016). Hence the absence of one component from this regulatory network might be maintained by other means. In this regard it is important to notice that Munc18c, i.e. the SM family protein involved in regulation of Sx4-containing SNARE complexes in response to insulin, can also bind to Sx2 and Sx3, and can accelerate fusion catalysed by these syntaxins *in vitro* (Yu et al., 2013). Hence, it is possible that insulin-stimulated translocation of ectopic HA-GLUT4-GFP is mediated by this kind of mechanism, but with reduced efficiency. This might explain the only partial effects observed in previous studies, in which Sx4 function was inhibited. Similar arguments can also explain the observed reduction in secretion of adiponectin.

However, a second, not mutually exclusive, model of Sx4 function should be considered. We speculate that our data indicate that, in the absence of Sx4, GLUT4 is unable to efficiently access GSCs/IRVs and, instead, is localised to the recycling endosomes. Under these conditions, we would predict that ubiquitylation of GLUT4 were to result in its degradation and that its cellular levels were to decline – a phenotype reported, for example, upon knockdown of Rab14 (Reed et al., 2013), depletion of Sx16 and Sx6 (Perera et al., 2003; Proctor et al., 2006), depletion of tankyrase (Sadler et al., 2019) and knockdown of SORT1 (our unpublished results). The reduction in GLUT4 levels within GSCs/IRVs observed in our study (Fig. 2C,D) indicates that Sx4 does play a role in the intracellular trafficking of GLUT4. Previous studies identified an intracellular pool of Sx4 that exhibits a small but reproducible insulin-stimulated translocation to the plasma membrane. Sx4 has also been observed within GLUT4-positive intracellular vesicles (Millar et al., 1999; Olson et al., 1997; Volchuk et al., 1996) but the functional consequences of this remain largely unexplored. One interpretation of our data is that, as well as mediating effects at the plasma membrane, Sx4 may function to control aspects of intracellular GLUT4 trafficking hitherto unappreciated.

It is important to notice that data obtained from knockout cells do not rule out an important role for Sx4 in insulin-stimulated GLUT4 translocation (Kioumourtzoglou et al., 2014). It is possible that adaptive responses to Sx4 knockout arise, which – although responsive to insulin – are less sensitive. Further studies are clearly required to pinpoint the insulin-sensitive mechanism(s) that regulate Sx4 function, and the data presented here represent an inroad to this end. In particular, our data presented here reveal an important role for Sx4 in maintaining steady-state GLUT4 levels in 3T3-L1 adipocytes and begin to define the molecular machinery that underpins adipocytokine secretion.

## MATERIALS AND METHODS

### 3T3-L1 cell culture and CRISPR genome editing

Mouse embryo fibroblast-derived 3T3-L1 adipocytes, purchased from the American Tissue Culture Collection (#CL-173) were grown, maintained and differentiated exactly as outlined previously (Roccisana et al., 2013; Sadler et al., 2015), and confirmed to be free from mycoplasma by routine testing. Cells were used between 10 and 14 days after induction of differentiation, and were serum-starved for 2 h prior to all assays.

A CRISPR/Cas9 plasmid, targeting exon 2 of the Sx4 gene (Target ID: MM0000265990, targeting site 5′-GAGGTTCGAGTCGCGCTGGTGG-3′) was purchased from Sigma Aldrich in the vector U6gRNA-Cas9-2A-RFP. The single vector expressed single guide RNA (sgRNA), Cas9 nuclease and RFP. 3T3-L1 cells were transfected using GeneCellin as per manufacturer's instructions. 24 h post transfection cells were trypsinised and pelleted at 800 rpm. Transfected cells were identified based on the presence of RFP fluorescence by sorting using a Beckman Coulter MoFlo Astrios flow cytometer and Summit 6.2 software. RFP-positive cells were serially diluted, plated in 10 cm dishes and monitored for the growth of single colonies.

Once formed, single-cell colonies were isolated by trypsinisation in colony rings. Colonies were expanded and Sx4 expression was screened using Immunoblotting and DNA sequencing. For sequencing, the genomic region surrounding the sgRNA-targeting site was amplified using the following primers; Forward 5′-GCAACTGTCGCCAATGACTC-3′, Reverse 5′-GAGGGGTTCCTCCTGAGACT-3′. Gel-purified PCR products were sequenced using the Eurofins Genomics' TubeSeq service, using the same primers. Sequences obtained from Sx4-knockout cells were compared to those from the equivalent DNA region amplified from 3T3-L1 wild-type cells.

### Oil Red O staining

Cells grown on coverslips were washed in phosphate-buffered saline, fixed using paraformaldehdye for 20 min then washed in 60% isopropanol before the addition of 0.05% Oil Red O stain in isopropanol. Cells were incubated in Oil Red O for 15 min, then rinsed in 60% isopropanol and photographed.

### Subcellular fractionation and immunoblotting

Adipocytes were homogenised and subjected to a subcellular fractionation protocol by successive centrifugation at 1000 ***g*** to remove large debris and intact cells; some of the plasma membrane is also present within this 1k P fraction. This was followed by centrifugation at 16,000 ***g***; the Supernatant from this fraction (16k S) has been shown to be enriched in GLUT4-containing vesicles, i.e. GSCs (Sadler et al., 2016a,b), whereas its pellet (16k P) contains dense membranes, including plasma membranes. Samples were resuspended in buffer for protein determination, and analysed by SDS-PAGE and immunoblotting exactly as described previously (Sadler et al., 2015). Immunoblot signals were quantified exactly as outlined by Sadler et al. (2015). Data shown are from a minimum of three biological replicates for each antibody.

### Antibodies

Anti-Sx4 (#110042), anti-Sx2 (#110022), anti-Sx3 (#110032), anti-Sx6 (#110062) and anti-Sx16 (#110161) were from Synaptic Systems (Göttingen, Germany) and were all used at 1:1000. Anti-GLUT1 (#652) and anti-GLUT4 (#654) were from Abcam (Cambridge, UK) (both used at 1:1000) and anti-GAPDH (#4300; used at 1:80,000) was from Ambion (Foster City, CA, USA). Detection antibodies were from LI-COR Biosciences (Lincoln, NE, USA) and were used at 1:10,000. Anti-sortilin (#12369-1-AP; 1:1000), anti-tankyrase (#18030-1-AP; 1:250) and anti-USP25 (#12199-1-AP; 1:1000) were from Proteintech. Anti-IRAP was from Paul Pilch (Boston College, Boston, MA, USA; 1:500). Anti-ACRP30 was as described previously (Clarke et al., 2006; 1:1000).

### Glucose transport

The accumulation of 2-deoxy-D-glucose (2DG) was assayed exactly as outlined (Roccisana et al., 2013). In these assays, the final concentration of 2DG was 50 µM and an uptake time of 5 min was employed unless otherwise stated. Assays were performed in triplicates; non-specific association of 2DG with the cells was determined using parallel assays containing 10 µM cytochalasin B. Each experiment was replicated on at least three biological repeats of cells; in the case of Sx4-knockout cells, data were validated using two independent isolates of Sx4-knockout cells. For assays using the selective GLUT inhibitor BAY-876 (Sigma) (Siebeneicher et al., 2016), cells were incubated with BAY-876 at 20 nM or 2 μM for 20 min prior to the addition of 2DG to initiate transport.

### HA-GLUT4-GFP recycling and FACS analysis

Kinetics analysis of GLUT4 trafficking was as previously described (Brewer et al., 2016). Briefly, 3T3-L1 fibroblasts were infected with lentivirus encoding haemagglutinin (HA)-tagged GLUT4 conjugated to GFP (HA-GLUT4-GFP) as described previously (Muretta et al., 2008). At the titres used, 70–80% of cells were infected and expressed the reporter protein; under these conditions, most of the infected cells are infected with only one virion (Muretta et al., 2008). Cells were differentiated into adipocytes as described above, using multi-well tissue culture plates. After labelling, cells were placed on ice, removed from the plates by collagenase digestion and single-cell fluorescence was analysed by flow cytometry. Insulin-sensitive, lipid droplet-filled adipocytes were identified based on light scatter (forward and side scatter) and cellular autofluorescence (488 excitation/>670 emission). Infected cells were distinguished from uninfected cells by using GFP fluorescence; uninfected cells in each sample were used as internal controls to measure background fluorescence from non-specific antibody labelling and autofluorescence. Similar levels of GFP fluorescence were measured in wild-type and Sx4-knockout cells, indicating that similar levels of HA-GLUT4-GFP expression was achieved in both cell types.

For all experiments, cells were serum-starved for 2 h at 37°C in low-serum medium [LSM; i.e. 0.5% FBS in Dulbecco's modified Eagle medium (DMEM)]. Insulin was added to LSM for the final 45 min of starvation, or for increasing times as indicated in the figure legends. To label surface GLUT4, cells were placed on ice and incubated with 25 μg/ml HA.11 monoclonal antibody (anti-HA; Covance) conjugated to Alexa-Fluor 647 (AF647-anti-HA) for 1 h (Brewer et al., 2016; Muretta et al., 2008). To measure GLUT4 uptake/recycling kinetics, cells were serum-starved in LSM supplemented with or without insulin and then incubated at 37°C in the continuous presence of AF647-anti-HA in LSM supplemented with or without insulin for increasing periods of time (from 0 to 65 minutes). To estimate the rate constant of endocytosis, cells were pre-treated with insulin for 45 min as described above, followed by treatment with wortmannin for increasing periods of time (from 0 to 60 minutes). GLUT4 remaining on the cell surface was then measured.

### Adiponectin assays

For analysis of secreted proteins, adipocytes were washed gently four times with serum-free DMEM then incubated in serum-free medium for 24 h. The next day, the medium was carefully harvested, centrifuged at 50,000 ***g*** for 30 min and the supernatant used in ACRP30 Quantikine ELISA assay (#MRP300; from R&D Systems Inc.).

### Statistical analysis

Statistical analyses were performed with GraphPad Prism 7. Where appropriate, the relevant statistical test that was implemented is reported in the figure legends. The level of significance was *P*=0.05.

## Supplementary Material

Supplementary information

Reviewer comments
