# Supplementary material for: Knockout of syntaxin-4 in 3T3-L1 adipocytes reveals new insight into GLUT4 trafficking and adiponectin secretion
Source: J Cell Sci. 2022 Jan 10;135(1):jcs258375. doi: 10.1242/jcs.258375 (PMC8767277; doi:10.1242/jcs.258375)
Supplement: Supplementary information [file joces-135-258375-s1.pdf]

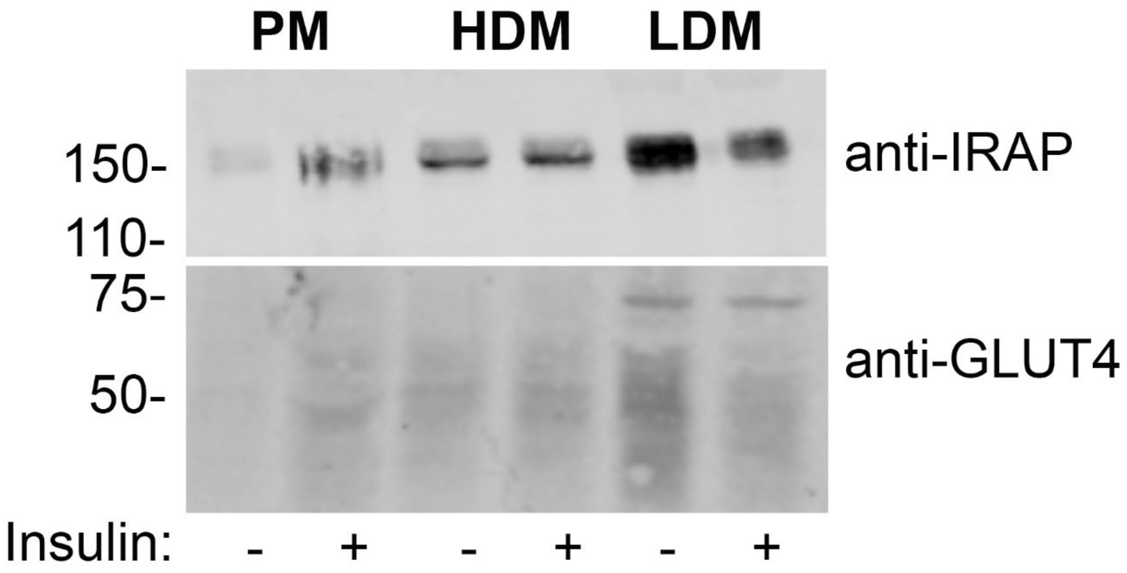

Fig. S1. IRAP exhibits insulin-stimulated translocation in Sx4 knockout cells.

Sx4 knockout adipocytes were incubated with or without 100 nM insulin for 20 minutes prior to subcellular fractionation as outlined (Sadler et al, 2015). Fractions enriched in plasma membranes (PM), high density microsomes (HDM) and low density microsomes (LDM) were generated, and equal fractions separated on SDS-PAGE and immunoblotted for levels of IRAP and GLUT4 as indicated. The data show that IRAP exhibits robust translocation from the LDM fraction to the PM fraction in response to insulin. Data shown from a representative experiment, replicated three times with qualitatively similar results. The approximate position of molecular weight markers is presented (in kDa).

Sadler, J.B.A., Bryant, N.J., and Gould, G.W. (2015). Characterization of VAMP isoforms in 3T3-L1 adipocytes: implications for GLUT4 trafficking. Mol. Biol. Cell *26*, 530–536.

Journal of Cell Science • Supplementary information
