# Supplementary material for: Knockout of syntaxin-4 in 3T3-L1 adipocytes reveals new insight into GLUT4 trafficking and adiponectin secretion
Source: J Cell Sci. 2022 Jan 10;135(1):jcs258375. doi: 10.1242/jcs.258375 (PMC8767277; doi:10.1242/jcs.258375)
Supplement: Reviewer comments [file joces-135-258375_review_history.pdf]

Knockout of syntaxin-4 in 3T3-L1 adipocytes reveals new insight into GLUT4 trafficking and adiponectin secretion.

Hannah L. Black, Rachel Livingstone, Cynthia C. Mastick, Mohammed Al Tobi, Holly Taylor, Angéline Geiser, Laura Stirrat, Dimitrios Kioumourtzoglou, John R. Petrie, James G. Boyle, Nia J. Bryant and Gwyn W. Gould
DOI: 10.1242/jcs.258375

Editor: Jennifer Lippincott-Schwartz

Review timeline

Original submission:	6 January 2021
Editorial decision:	18 February 2021
First revision received:	22 September 2021
Editorial decision:	27 October 2021
Second revision received:	10 November 2021
Accepted:	18 November 2021

Original submission

First decision letter

MS ID#: JOCES/2021/258375

MS TITLE: Knockout of Syntaxin-4 in 3T3-L1 adipocytes reveals new insight into GLUT4 trafficking and adiponectin secretion.

AUTHORS: Hannah L Black, Rachel Livingstone, Cynthia C Mastick, Mohammed Al Tobi, Angéline Geiser, Laura Stirrat, John R Petrie, James G Boyle, Nia J Bryant, and Gwyn W Gould
ARTICLE TYPE: Research Article

We have now reached a decision on the above manuscript.

To see the reviewers' reports and a copy of this decision letter, please go to: https://submit-jcs.biologists.org and click on the 'Manuscripts with Decisions' queue in the Author Area. (Corresponding author only has access to reviews.)

As you will see, the reviewers raise a number of substantial criticisms that prevent me from accepting the paper at this stage. They suggest, however, that a revised version might prove acceptable, if you can address their concerns. The criticisms of your paper are numerous, so I expect you will need to perform many new experiments to address them. If you think that you can deal satisfactorily with the criticisms on revision, I would be pleased to see a revised manuscript. We would then return it to the reviewers.

We are aware that you may be experiencing disruption to the normal running of your lab that makes experimental revisions challenging. If it would be helpful, we encourage you to contact us to discuss your revision in greater detail. Please send us a point-by-point response indicating where you are able to address concerns raised (either experimentally or by changes to the text) and where you will not be able to do so within the normal timeframe of a revision. We will then provide further guidance. Please also note that we are happy to extend revision timeframes as necessary.

© 2021. Published by The Company of Biologists under the terms of the Creative Commons Attribution License (https://creativecommons.org/licenses/by/4.0/).

Please ensure that you clearly highlight all changes made in the revised manuscript. Please avoid using 'Tracked changes' in Word files as these are lost in PDF conversion.

I should be grateful if you would also provide a point-by-point response detailing how you have dealt with the points raised by the reviewers in the 'Response to Reviewers' box. Please attend to all of the reviewers' comments. If you do not agree with any of their criticisms or suggestions please explain clearly why this is so.

Reviewer 1

Advance summary and potential significance to field

Black et al report that knock out of Sx4 in 3T3-L1 adipocytes by CRISPR decreases Glut4 expression and reduces the rate of Glut4 exocytosis. Adiponectin secretion is also diminished.

Comments for the author

The Glut4-related part of the study confirms a large body of literature showing that Sx4 is not absolutely required for Glut4 translocation. This is not surprising given a significant redundancy in the biological activity of various SNAREs. At this point, it seems important to figure out what t-SNARE(s) replaces Sx4 in the process of fusion of Glut4-vesicles, and the authors are in a good position to address this problem.
The effect of Sx4 KO on the expression of Glut4 is interesting but preliminary. What is the explanation for this effect? Does Glut4 mRNA go down? Is stability of Glut4 affected? In sum, I feel that the whole study is very preliminary and does not contain a sufficient amount of novel data.

Reviewer 2

Advance summary and potential significance to field

In this study the authors examine the role of a syntaxin-4 in insulin regulation of GLUT4 traffic. Despite this question having been previously examined in both cultured adipocytes and muscle-like cells using knockdown and dominant-inhibitory approaches, and in heterozygous knockout mice, no clear picture for the role of syntaxin-4 in GLUT4 transport has emerged. In aggregate, the literature supports significant redundancy in snare requirement for GLUT4 translocation, and although the data support a role for syntaxin-4, the effect sizes are smaller than would be expected if syntaxin-4 was the snare required for GLUT4-containing vesicles fusion with the plasma membrane.

Here the authors use knockout approaches to examine the effects of silencing syntaxin-4 in cultured adipocytes on GLUT4 and adiponectin. They report syntaxin-4 knockout reduces insulin-stimulated glucose transport, cellular GLUT4 levels and adiponectin secretion by 30 to 50%. Thus, their results are in agreement with those of the prior studies. Although this work is well performed and the data generally support the conclusions, the study fails to address the mechanism(s) of how syntaxin-4 knockout is linked to reduced cellular GLUT4 levels or adiponectin secretion. In its current form the manuscript, although raising numerous interesting possibilities for mechanism, is significantly underpowered by experimental data exploring those possibilities.

Comments for the author

In its current form the manuscript, although raising numerous interesting possibilities for mechanism, is significantly underpowered by experimental data exploring those possibilities. Specific comments are listed below.
1. All the bar graphs should show the individual data points to capture the number of experiments and how the data are scattered. Simple SD and SEM for such small numbers is not sufficient.
2. There are too many grammatical errors and poorly constructed sentences, making it difficult to follow or understand the points. The authors should carefully check the manuscript.

© 2021. Published by The Company of Biologists under the terms of the Creative Commons Attribution License (https://creativecommons.org/licenses/by/4.0/).

3. The authors hypothesize that syntaxin-4 depletion leads to reduction in cellular GLUT4 levels. It will be crucial to have a mechanistic link to validate their claim. The authors suggest enhanced ubiquitination of GLUT4 as a possible cause. Why not test this by examining ubiquitination of GLUT4 levels in knockout and wild-type cells? Also, why not use a lysosomal or a proteosome inhibitor to see if there is a rescue of the reduced GLUT4 levels in the knockout cells?
4. The authors state that 'the reduction in adiponectin secretion arises from defective exocytosis of the protein rather than an effect on its expression or stability.' This analysis is quite incomplete. Is adiponectin released into the media during 24 hrs incubation in serum-free culture conditions an appropriate test of the hypothesis for the role of syntaxin-4 in regulation of adiponectin secretion? What about leptin or adipsin? What about controls for recycling of membrane proteins such as LDL or TR?
5. The authors have used subcellular fractionation to investigate the effects of syntaxin-4 depletion on the cellular distributions of other SNAREs and other proteins involved in the GLUT4 trafficking pathway.
These analyses would be significantly augmented by assessing colocalization using immunolocalization in microscopy.
6. The authors claim that insulin signaling is not impaired by syntaxin-4 depletion as evident from the similar EC50 values for insulin in 2DG uptake in Fig 3. This claim needs to be supported by assessing the levels of phospho-Akt, phospho-TBC1D4 and insulin receptor.
7. The authors hypothesize that syntaxin-4 depletion possibly results in mis-localization of GLUT4 to the recycling endosomes instead of the GSVs. They present no data to support this statement. One could argue this is the key conclusion of the work. This claim needs to be supported by data that tests the hypothesis. And as noted above, they also need to determine the effect of syntaxin-4 knockout on recycling endosome cargoes.
8. Have the authors determined if syntaxin-4 depletion affects cellular GLUT1 levels? Also, why do they think a reduction in GLUT1 level is observed in the GSVs containing fraction (Fig 3C) in the syntaxin-4-
knockout cells?
9. Can the authors explain why in Figure 4B, they do not observe a change in Sx6 levels in any of the cellular fractions, though the total cellular level of syntaxin-6 is significantly reduced in the syntaxin-6-
knockout cells?
10. Do the syntaxin-4 levels increase or decrease with differentiation in 3T3-WT cells? The blots for syntaxin-4 and anti-tubulin should be replaced with better blots to understand the same in Fig 1B.
11. In Fig 6B, syntaxin-4 KO results in 30% depletion of surface GLUT4 levels when GLUT4 is expressed ectopically. The authors should provide a western blot here comparing the levels of the ectopically expressed GLUT4 in the wild-type and KO cell lines.
12. In Fig 2B, the * sign for $p<0.02$ should be provided on the bars for better understanding.
13. In the figure legend of Fig 3, the authors should be consistent with their use of 2DG or DG and not both, as it is confusing.

Reviewer 3

Advance summary and potential significance to field

Dr. Gould et al. investigated the function of Syntaxin-4 in 3T3-L1 adipocytes in this study. They knocked out Syntaxin-4 using CRISPR-Cas9 in 3T3-L1 cells and then examined the roles of Syntaxin-4 in adiponectin secretion and GLUT4 trafficking. It is demonstrated that Syntaxin-4 knockout led to decreased adiponectin secretion and accumulation inside cells, as well as reduced GLUT4 level, preferentially in GLUT4 storage vesicles. Overall, these experiments are well designed, data are presented clearly, and discussion is very thoughtful.

Comments for the author

1. IRAP and GLUT4 co-localized well in 3T3-L1 adipocytes, indicating the two proteins take identical trafficking routes. Does the reduction of GLUT4, but not IRAP, in GSVs suggest Syntaxin-4 plays specific roles in the sorting of GLUT4 into GSVs? Can insulin-stimulated IRAP translocation be

© 2021. Published by The Company of Biologists under the terms of the Creative Commons Attribution License (https://creativecommons.org/licenses/by/4.0/).

evaluated in Syntaxin-4 knockout cells to confirm that insulin-responsiveness of GSVs and of IRAP are still normal in these cells?
2. Meanwhile, some rescue experiments with Syntaxin-4 re-expressed in the knockout line will help to establish the specificity of Syntaxin-4 knockout by CRISPR-Cas9.

First revision

Author response to reviewers' comments

Thank you for your patience and understanding of the time it has taken us to carry out the agreed revisions to our manuscript **Black et al. 'Knockout of Syntaxin-4 in 3T3-L1 adipocytes reveals new insight into GLUT4 trafficking and adiponectin secretion.'**

Below is a detailed description of how we have addressed those comments that we agreed needed to be actioned (as per email exchange of 24th/25th of February 2021); reviewers comments are in red, with our responses in black. For completion we have included our response to all comments at the end (as sent in our email 24/02/2021).

Reviewer-1.
It was agreed no suggestions needed to be addressed.

Reviewer-2 (addressing comments that we agreed needed to be addressed in the manuscript).
All the bar graphs should show the individual data points to capture the number of experiments and how the data are scattered. Simple SD and SEM for such small numbers is not sufficient.
We have changed all the bar graphs for the immunoblot data, Figures 2, 3, 4 and 5 to present the values of each individual experiment. 'Small numbers' is difficult to understand. We report statistically significant changes determined by appropriate statistical tests widely employed in this field, and the level of accuracy and variability is clearly reproduced.

The authors state that 'the reduction in adiponectin secretion arises from defective exocytosis of the protein rather than an effect on its expression or stability.' This analysis is quite incomplete. Is adiponectin released into the media during 24 hrs incubation in serum-free culture conditions an appropriate test of the hypothesis for the role of syntaxin-4 in regulation of adiponectin secretion? What about leptin or adipsin? What about controls for recycling of membrane proteins such as LDL or TR?
As discussed in XXX, there is a lot to unpack here, and we agreed that doing so would not add to the manuscript and would introduce further complications beyond the nature of this report. However, we have added a discussion on this issue on page 7.

The authors claim that insulin signaling is not impaired by syntaxin-4 depletion as evident from the similar EC50 values for insulin in 2DG uptake in Fig 3. This claim needs to be supported by assessing the levels of phospho-Akt, phospho-TBC1D4 and insulin receptor.
We agreed that this important point needed to be examined. We present a completely new Figure 1, which is discussed further below showing robust insulin-stimulated phosphorylation of Akt and ERK in wild-type and syntaxin-4 knockout adipocytes.

Do the syntaxin-4 levels increase or decrease with differentiation in 3T3-WT cells? The blots for syntaxin-4 and anti-tubulin should be replaced with better blots to understand the same in Fig 1B.
The new Figure 1B addresses this point and includes higher quality blots as requested. However, we are wary of making a definitive statement about whether Sx4 levels increase or decrease upon differentiation because of the considerably different size of 3T3-L1 fibroblasts and adipocytes. This is well illustrated by inspection of the GAPDH immunoblots shown in the new figure 1B where we have loaded equivalent protein levels in each lane.

In Fig 6B, syntaxin-4 KO results in 30% depletion of surface GLUT4 levels when GLUT4 is expressed ectopically. The authors should provide a western blot here comparing the levels of the ectopically expressed GLUT4 in the wild-type and KO cell lines.

© 2021. Published by The Company of Biologists under the terms of the Creative Commons Attribution License (https://creativecommons.org/licenses/by/4.0/).

As discussed previously, we do not routinely measure HA-GLUT4-GFP levels by immunoblotting in these experiments as we aim for very low levels of expression. We focussed on obtaining a level of expression sufficient for ready identification by FACS, but not so large as to 'swamp' GLUT4 sorting machinery. To this end, we used viral titres which have been previously established (by CMM). These conditions have been well-described as cited in the manuscript, (e.g. Brewer et al., Muretta et al., Muretta and Mastick, Habtemichael et al., Reed et al.). Using FACS quantification, the levels of GFP (a read out of HA-GLUT4-GFP expression) were similar between the two cell types, a point now made in both *Materials and Methods* and the legend to Figure 6.

In Fig 2B, the * sign for $p<0.02$ should be provided on the bars for better understanding.
Corrected.

In the figure legend of Fig 3, the authors should be consistent with their use of 2DG or DG and not both, as it is confusing.
Corrected.

Reviewer 3 (addressing comment that we agreed needed to be addressed in the manuscript). IRAP and GLUT4 co-localized well in 3T3-L1 adipocytes, indicating the two proteins take identical trafficking routes. Does the reduction of GLUT4, but not IRAP, in GSVs suggest Syntaxin-4 plays specific roles in the sorting of GLUT4 into GSVs? Can insulin-stimulated IRAP translocation be evaluated in Syntaxin-4 knockout cells to confirm that insulin-responsiveness of GSVs and of IRAP are still normal in these cells?
This is an interesting question. However, it is not a straightforward one to answer. The Syntaxin 4 knockout cells used here still express endogenous GLUT4 at a level roughly half of that seen in wild- type cells. It is therefore unclear whether this reduction in GLUT4 would necessarily equate to a significant reduction in IRAP translocation. This is made even more difficult as measurement of IRAP translocation is limited to subcellular fractionation-based approaches. Nevertheless, we performed subcellular fractionation of Sx4 knockout adipocytes and have observed consistent insulin- stimulated IRAP translocation in these cells. We are however reluctant to include these data in the main body of the manuscript as they cannot be quantified robustly (due to very low levels of IRAP in the PM fraction under basal conditions) and have instead included them as Supplemental Figure 1. It is, however, important to note that we have carefully quantified insulin-dependent changes cell surface GLUT4 levels in control and Sx4 knockout adipocytes which support the idea that these cells retain insulin-responsive trafficking mechanisms.

RESPONSE to ALL COMMENTS (sent 24/02/2021, agreed by Professor Lippincott-Schwartz)

Black et al.
Knockout of Syntaxin-4 in 3T3-L1 adipocytes reveals new insight into GLUT4 trafficking and adiponectin secretion.
Thank you for the reviews of our paper. We would like to dissect the issues raised and ask if our proposed response/planned additional experiments, would in your opinion constitute an appropriate response. Our laboratories are working at less than 50% occupancy as a result of social distancing measures and hence our capacity for further experimental work is severely limited. This is unlikely to change in the near future. Below, reviewers comments are in red, with our comments in black.

Reviewer-1.

This reviewer suggests our work 'confirms a large body of literature showing that Sx4 is not absolutely required for GLUT4 translocation'. We would respectfully point out that ours is the only study in which absolute knockout of Sx4 is achieved. Homozygous Sx4 knockout mice are lethal, hence all work has been performed in heterozygotes, and all work in cell lines uses either incomplete siRNA knockdown (not knockout) or makes use of dominant negative protein domains. While we do not dispute the utility of these models, as we clearly note in our introduction, there are limitations of the published studies which our knockout system overcomes. Hence to dismiss this in this manner is a little dismaying. We do not believe this reviewer has made any constructive suggestions that we should address.

© 2021. Published by The Company of Biologists under the terms of the Creative Commons Attribution License (https://creativecommons.org/licenses/by/4.0/).

Reviewer-2.
This reviewer notes: 'Despite this question having been previously examined in both cultured adipocytes and muscle-like cells using knockdown and dominant-inhibitory approaches, and in heterozygous knockout mice, no clear picture for the role of syntaxin-4 in GLUT4 transport has emerged.' We agree. As noted above, the lack of a clear picture is in part a consequence of the systems employed (knockdown versus knockout; heterozygous mice; limited assay sensitivity compared to our kinetic analysis using HA-GLUT4-GFP recycling, etc.). We do not see this as a weakness, rather it underscores the novelty and importance of our work.

'…although the data support a role for syntaxin-4, the effect sizes are smaller than would be expected if syntaxin-4 was the snare required for GLUT4-containing vesicles fusion with the plasma membrane.' We agree; the latter statement encapsulates the key conclusion of our study. This conclusion cannot be drawn from any of the published studies.

'…the study fails to address the mechanism(s) of how syntaxin-4 knockout is linked to reduced cellular GLUT4 levels or adiponectin secretion.' We have reported what we believe to be a well-powered study to reveal effects of Sx4 knockdown on GLUT4 trafficking. Importantly, we show that the capacity for GLUT4 translocation is retained in Sx4 knockdown cells, we show that this is not a consequence of up-regulated plasma membrane SNARE expression, dissect at what steps in the recycling kinetics this takes place and show that the plasma membrane fusion machinery is largely intact. No previous study can make these conclusions as in all previous analyses, either residual Sx4 protein remained after knockdown, or its function was not completely inhibited, or in the case of the transgenic mice the animals are heterozygous.

This reviewer has, we believe, made a decision on perceived 'impact' and then proceeded to justify it with a series of bullet-point suggestions. Some of these make reasonable points, others we argue do not. We respond to these numerically as detailed in the report.

Reviewer 2 Comments for the Author:
In its current form the manuscript, although raising numerous interesting possibilities for mechanism, is significantly underpowered by experimental data exploring those possibilities. We do not understand what is meant by 'underpowered'. All data have been analysed using statistical approaches validated and widely used in the field, and with what we believe to be appropriate methods.

Specific comments are listed below.
1. All the bar graphs should show the individual data points to capture the number of experiments and how the data are scattered. Simple SD and SEM for such small numbers is not sufficient.
This is a simple fix and we will present the data in this manner. 'Small numbers' is difficult to understand. We report statistically significant changes and the level of accuracy and variability is clearly reproduced.

2. There are too many grammatical errors and poorly constructed sentences, making it difficult to follow or understand the points. The authors should carefully check the manuscript.
Again – a somewhat strange statement. We cannot find any examples of poor grammar, but if there are we would be delighted to address them. Sadly, no examples are provided.

3. The authors hypothesize that syntaxin-4 depletion leads to reduction in cellular GLUT4 levels. It will be crucial to have a mechanistic link to validate their claim. The authors suggest enhanced ubiquitination of GLUT4 as a possible cause. Why not test this by examining ubiquitination of GLUT4 levels in knockout and wild-type cells? Also, why not use a lysosomal or a proteosome inhibitor to see if there is a rescue of the reduced GLUT4 levels in the knockout cells?
This set of comments has missed a key point - we cannot measure increased ubiquitination of a protein that has been degraded. For this reason, we speculated on the potential mechanism in the discussion section as to test this hypothesis would be extraordinarily challenging. Similarly, proteosomal inhibitors are well-known to change GLUT4 levels in control cells – unravelling these effects will be far from straightforward in a cell in which GLUT4 levels are already decreased and would add little to the central conclusion of our work.

© 2021. Published by The Company of Biologists under the terms of the Creative Commons Attribution License (https://creativecommons.org/licenses/by/4.0/).

4. The authors state that 'the reduction in adiponectin secretion arises from defective exocytosis of the protein rather than an effect on its expression or stability.' This analysis is quite incomplete. Is adiponectin released into the media during 24 hrs incubation in serum-free culture conditions an appropriate test of the hypothesis for the role of syntaxin-4 in regulation of adiponectin secretion? What about leptin or adipsin? What about controls for recycling of membrane proteins such as LDL or TR?
There is a lot to unpack here.
First, measuring adiponectin secretion into the media over 24 has revealed a clear reduction in secretion. We are not clear what is 'inappropriate' about this widely used approach. We do not understand what the reviewer takes issue with on this experiment.
We can certainly examine effects on other adipocytokine release patters, such as leptin, ZAG and members of the IL family as reagents for these are available in our group. However, given that the mechanism and patterns of adipocytokines vary, we are not clear where this analysis will go: we may see parallel changes in other proteins or quite distinct patters. Will this add to the manuscript?

5. The authors have used subcellular fractionation to investigate the effects of syntaxin-4 depletion on the cellular distributions of other SNAREs and other proteins involved in the GLUT4 trafficking pathway. These analyses would be significantly augmented by assessing colocalization using immunolocalization in microscopy.
Total levels of proteins and their distribution among subcellular fractions has revealed no changed in any cell surface SNARE, Sx16 or the cargo protein IRAP in Sx4 knockdown cells. We fail to understand what information immunofluorescence would add. If this reviewer is implying that more subtle effects may be revealed by looking at overlap with GLUT4 (which is what we think 'colocaoisation' implies) these will be hard to quantify given that GLUT4 levels are markedly reduced in the Sx4 knockout cells.

6. The authors claim that insulin signaling is not impaired by syntaxin-4 depletion as evident from the similar EC50 values for insulin in 2DG uptake in Fig 3. This claim needs to be supported by assessing the levels of phospho-Akt, phospho-TBC1D4 and insulin receptor.
We have data showing robust insulin-stimulated phosphorylation of Akt and Insulin Receptor and can include this in a revised version of the paper.

7. The authors hypothesize that syntaxin-4 depletion possibly results in mis-localization of GLUT4 to the recycling endosomes instead of the GSVs. They present no data to support this statement. One could argue this is the key conclusion of the work. This claim needs to be supported by data that tests the hypothesis. And as noted above, they also need to determine the effect of syntaxin-4 knockout on recycling endosome cargoes.
Our speculation that mis-sorting of GLUT4 into recycling endosomes may result in enhanced degradation is confined to the Discussion where we consider potential mechanisms for the data we have described. There are two points germane to this. First, it is hard to identify how we could quantify enhanced GLUT4 localisation to recycling endosomes when GLUT4 protein levels are markedly reduced. Quantifying overlap of GLUT4 with (say) endosomal markers is difficult at the best of times in adipocytes with small cytosolic volumes and many fat droplets. Doing so when GLUT4 levels are reduced is even more challenging and we do not believe we have the capability t measure this with the accuracy required to make a firm conclusion. Secondly, this is not a parameter that is widely used in the GLUT4 field precisely because it is difficult to measure accurately.
We note that this is, in our opinion, far from the 'key conclusion of the work.

8. Have the authors determined if syntaxin-4 depletion affects cellular GLUT1 levels? It is shown. Also, why do they think a reduction in GLUT1 level is observed in the GSVs containing fraction (Fig 3C) in the syntaxin-4-knockout cells? Yes, data on GLUT1 levels is presented in Figure 3. The reviewer then asks why GLUT1 levels fall in the GSV-enriched fraction. We note that a trend towards a reduction in whole cell lysate levels of GLUT1 is observed. By the variation in this experimental set was large and did not reach significance. This reduction was observed in the 16kS fraction.

9. Can the authors explain why in Figure 4B, they do not observe a change in Sx6 levels in any of the cellular fractions, though the total cellular level of syntaxin-6 is significantly reduced in the

© 2021. Published by The Company of Biologists under the terms of the Creative Commons Attribution License (https://creativecommons.org/licenses/by/4.0/).

syntaxin- 6-knockout cells? There is a degree of variability in this dataset. The values do not reach significance in the subcellular fractions presumably as a consequence of this.

10. Do the syntaxin-4 levels increase or decrease with differentiation in 3T3-WT cells? The blots for syntaxin-4 and anti-tubulin should be replaced with better blots to understand the same in Fig 1B. We will produce a new set of immunoblots to reveal these differences more clearly.

11. In Fig 6B, syntaxin-4 KO results in 30% depletion of surface GLUT4 levels when GLUT4 is expressed ectopically. The authors should provide a western blot here comparing the levels of the ectopically expressed GLUT4 in the wild-type and KO cell lines.
We do not routinely measure HA-GLUT4-GFP levels by immunoblotting in these experiments. We focussed on obtaining a very low level of expression, sufficient for ready identification by FACS, not so large as to 'swamp' GLUT4 sorting machinery, using a set of viral titres which have been previously established. These conditions have been well-described by many groups. Using FACS quantification, the levels of GFP (a read out of HA-GLUT4-GFP expression) were similar between the two cell types. We can make this clear in a revision.

12. In Fig 2B, the * sign for $p<0.02$ should be provided on the bars for better understanding. We will correct this.

13. In the figure legend of Fig 3, the authors should be consistent with their use of 2DG or DG and not both, as it is confusing.
We will correct this.

Reviewer 3 Advance Summary and Potential Significance to Field:
Dr. Gould et al. investigated the function of Syntaxin-4 in 3T3-L1 adipocytes in this study. They knocked out Syntaxin-4 using CRISPR-Cas9 in 3T3-L1 cells and then examined the roles of Syntaxin-4 in adiponectin secretion and GLUT4 trafficking. It is demonstrated that Syntaxin-4 knockout led to decreased adiponectin secretion and accumulation inside cells, as well as reduced GLUT4 level, preferentially in GLUT4 storage vesicles. Overall, these experiments are well designed, data are presented clearly, and discussion is very thoughtful.
We thank this reviewer for a constructive set of comments.

Reviewer 3 Comments for the Author:
1. IRAP and GLUT4 co-localized well in 3T3-L1 adipocytes, indicating the two proteins take identical trafficking routes. Does the reduction of GLUT4, but not IRAP, in GSVs suggest Syntaxin-4 plays specific roles in the sorting of GLUT4 into GSVs? Can insulin-stimulated IRAP translocation be evaluated in Syntaxin-4 knockout cells to confirm that insulin-responsiveness of GSVs and of IRAP are still normal in these cells?
This is a valid point. We will perform these experiments.

2. Meanwhile, some rescue experiments with Syntaxin-4 re-expressed in the knockout line will help to establish the specificity of Syntaxin-4 knockout by CRISPR-Cas9.
This is challenging in these cells. The ability to differentiate into adipocytes is progressively diminished as passage number increases. We have found that the Sx4 knockout lines can only differentiate well for around 6 to 7 passages, hence generating a cell line which can stabley re-express Sx4 has been problematic and we have been unable to achieve this in a cell line that can retain differentiation capacity.

Second decision letter

MS ID#: JOCES/2021/258375

MS TITLE: Knockout of Syntaxin-4 in 3T3-L1 adipocytes reveals new insight into GLUT4 trafficking and adiponectin secretion.

© 2021. Published by The Company of Biologists under the terms of the Creative Commons Attribution License (https://creativecommons.org/licenses/by/4.0/).

AUTHORS: Hannah L Black, Rachel Livingstone, Cynthia C Mastick, Mohammed Al Tobi, Angeline Geiser, Holly Taylor, Laura Stirrat, Dimitrios Kioumourtzoglou, John R Petrie, James G Boyle, Nia J Bryant, and Gwyn W Gould
ARTICLE TYPE: Research Article

Please address the few remaining issues raised by the reviewers and send back your manuscript to our office. I will then make a final decision regarding publication.

Please ensure that you clearly highlight all changes made in the revised manuscript. Please avoid using 'Tracked changes' in Word files as these are lost in PDF conversion.

I should be grateful if you would also provide a point-by-point response detailing how you have dealt with the points raised by the reviewers in the 'Response to Reviewers' box. Please attend to all of the reviewers' comments. If you do not agree with any of their criticisms or suggestions please explain clearly why this is so.

Reviewer 1

Advance summary and potential significance to field

I think that findings reported by Black et al are either confirmatory (the role of Sx4 in Glut4 exocytosis) or preliminary (Sx4 and Glut4 expression). What is the explanation for the latter effect? Does Glut4 mRNA go down? Is stability of Glut4 affected?

Comments for the author

Multiple papers done both in vivo and in vitro have shown, in agreement with their major conclusion, that Sx4 is not absolutely required for Glut4 translocation. Since CRISPR has not been used for knockout of Sx4 previously, I can understand that the authors do not see their results as confirmatory. It is less clear to me why they have not looked into decreased Glut4 levels in Sx4 KO cells. This observation seems interesting especially that IRAP does not seem to be affected. At the very least, they should determine if Glut4 mRNA and/or stability is decreased.

Reviewer 2

Advance summary and potential significance to field

The study is well performed and addresses an important (and controversial) aspect of the control of Glut4.
The study is, at least in my opinion, somewhat limited in scope, which blunts its overall impact. However, in the end it is the community that should decide the impact of the findings not the reviewers.
The major findings are that Syntaxin-4 knockout results in a modest reduction of Glut4 translocation to the plasma membrane and a larger decrease in glucose uptake, the latter likely due to a decrease in total Glut4 induced by Syntaxin-4 knockout. As discussed by the authors, there are a number of possibilities for how Syntaxin-4 knockout alters Glut4 and adiponectin. Those possibilities have not been explored in this study.

Comments for the author

I have no suggestions.
I feel I should justify my comment about the number of replicate. The point about replicate numbers is that the statistics (that we all use) were not developed to compare small numbers. I am sure the authors will agree that it is not possible to describe a normal distribution using 3 points. I agree that the authors have used field standard replicate numbers and statistics, and I did not mean to specifically criticize that aspect of the study. The comment on numbers of replicates was to justify why it is important to show the individual data points rather than to solely depend on p values.

© 2021. Published by The Company of Biologists under the terms of the Creative Commons Attribution License (https://creativecommons.org/licenses/by/4.0/).

Reviewer 3

Advance summary and potential significance to field

Dr. Gould et al. investigated the function of Syntaxin-4 in 3T3-L1 adipocytes in this study. They knocked out Syntaxin-4 using CRISPR-Cas9 in 3T3-L1 cells and then examined the roles of Syntaxin-4 in adiponectin secretion and GLUT4 trafficking. It is demonstrated that Syntaxin-4 knockout led to decreased adiponectin secretion and accumulation inside cells, as well as reduced GLUT4 level, preferentially in GLUT4 storage vesicles. Overall, these experiments are well designed, data are presented clearly, and discussion is very thoughtful.

Comments for the author

1. IRAP and GLUT4 co-localized well in 3T3-L1 adipocytes, indicating the two proteins take identical trafficking routes. Does the reduction of GLUT4, but not IRAP, in GSVs suggest Syntaxin-4 plays specific roles in the sorting of GLUT4 into GSVs? Can insulin-stimulated IRAP translocation be evaluated in Syntaxin-4 knockout cells to confirm that insulin-responsiveness of GSVs and of IRAP are still normal in these cells?
Supplementary Figure-1 now shows that the insulin responsiveness of IRAP remains unchanged in Syntaxin-4 knockout cells. The specific effects of Syntaxin-4 knockout on GLUT4 level and trafficking is well covered in the discussion section.
2. Meanwhile, some rescue experiments with Syntaxin-4 re-expressed in the knockout line will help to establish the specificity of Syntaxin-4 knockout by CRISPR-Cas9.
The authors noted the technical challenges with the experiments, which is agreeable given the finicky nature of 3T3-L1 cells.

Second revision

Author response to reviewers' comments

Reviewer 1 had raised the issue of our work being either confirmatory or preliminary, but we are pleased to see that they now seem satisfied with our response about why this is not the case.

This reviewer also asked “Does Glut4 mRNA go down? Is stability of Glut4 affected?” Questions regarding GLUT4 stability/mRNA levels are, to our mind, new avenues of investigation which would not be as straightforward or informative as the reviewer implies. For the experiments the reviewer suggests to be meaningful the analysis could not be limited to GLUT4 (we would need to consider GLUT1 and IRAP at least) and then delve far deeper into potential mechanisms to make sense of any results. This represents a significant amount of work and even if completed would leave us with an unknown mechanism of why GLUT4 levels are reduced. Yes, we would know if this is protein stability or mRNA stability effect, but this work would provide no mechanistic insight as to how Sx4 knockout might cause this and importantly would not add anything to the central message of our manuscript.

Reviewer 2 has no suggestions for us to address and is happy with our comments. We note their thoughts on statistical analysis and thank them for clarifying their meaning.

Reviewer 3 was very positive about our study (“Overall, these experiments are well designed, data are presented clearly, and discussion is very thoughtful.”). We addressed their questions in revision and as noted they believe the points to have been clarified and have not asked for further changes.

© 2021. Published by The Company of Biologists under the terms of the Creative Commons Attribution License (https://creativecommons.org/licenses/by/4.0/).

Third decision letter

MS ID#: JOCES/2021/258375

MS TITLE: Knockout of Syntaxin-4 in 3T3-L1 adipocytes reveals new insight into GLUT4 trafficking and adiponectin secretion.

AUTHORS: Hannah L Black, Rachel Livingstone, Cynthia C Mastick, Mohammed Al Tobi, Angeline Geiser, Holly Taylor, Laura Stirrat, Dimitrios Kioumourtzoglou, John R Petrie, James G Boyle, Nia J Bryant, and Gwyn W Gould
ARTICLE TYPE: Research Article

I am happy to tell you that your manuscript has been accepted for publication in Journal of Cell Science, pending standard ethics checks.

© 2021. Published by The Company of Biologists under the terms of the Creative Commons Attribution License (https://creativecommons.org/licenses/by/4.0/).
